# Seizures in congenital heart disease: risks, outcomes, and birth cohort data from China

**DOI:** 10.3389/fped.2026.1936201

**Published:** 2026-08-25

**Authors:** Li Wang, Lei Cao, Chuan Zhang, Wei Zhang

**Affiliations:** 1 Department of Pediatric Neurology, Gansu Provincial Maternity and Child Care Hospital (Gansu Provincial Central Hospital), Lanzhou, Gansu, China; 2 Gansu Provincial Clinical Research Center for Birth Defects and Rare Diseases, Gansu Provincial Maternity and Child-care Hospital (Gansu Provincial Central Hospital), Lanzhou, Gansu, China; 3 Department of Pediatric Cardiology, Nephrology and Rheumatology Immunology, Gansu Provincial Maternity and Child Care Hospital (Gansu Provincial Central Hospital), Lanzhou, Gansu, China

**Keywords:** birth cohort, congenital heart disease, electroencephalography, neurodevelopmental outcomes, seizures, single-ventricle physiology

## Abstract

**Objective:**

Congenital heart disease is the most common birth defect and carries a high risk of seizures; however, specific risk factors and long-term neurodevelopmental outcomes remain poorly defined. We aimed to identify factors associated with seizure occurrence and evaluate neurodevelopmental outcomes in children with congenital heart disease.

**Methods:**

This prospective cohort study included 31,734 singleton live births in Lanzhou, China (2018–2023) followed for 18 months. Maternal, demographic, and clinical data were prospectively collected. Congenital heart disease phenotypes were classified as left-to-right shunt, cyanotic, left-sided, or single-ventricle lesions. Univariate logistic regression was conducted to identify factors associated with clinical seizures, and electroclinical and neurodevelopmental profiles were systematically evaluated.

**Results:**

Congenital heart disease was diagnosed in 392 infants (1.2%), of whom 23 (5.9%) experienced clinical seizures. Lower birthweight, prolonged hospitalization, and extracorporeal membrane oxygenation support were significantly associated with seizure occurrence (all *P* < 0.001). Among congenital heart disease phenotypes, single-ventricle physiology had the highest seizure incidence (5.8%, *P* < 0.001). Electroencephalography identified focal impaired awareness seizures (*n* = 13), electrographic-only seizures (*n* = 2), and diverse interictal abnormalities. At 18 months, children with seizures demonstrated significantly lower motor scores (*P* = 0.038) and higher rates of global developmental delay/intellectual disability (*P* < 0.001).

**Conclusions:**

Seizures represent a critical complication in infants with congenital heart disease, particularly those with single-ventricle physiology or requiring prolonged hospitalization or mechanical circulatory support. Seizure development was associated with poorer 18-month neurodevelopmental outcomes, underscoring the need for proactive neurological surveillance and larger multicenter studies to identify independent risk pathways.

## Introduction

Congenital Heart Disease (CHD) is among the most prevalent structural birth defects worldwide, affecting approximately 1% of live births (1). Recent advances in pediatric cardiology and cardiothoracic surgery have dramatically improved survival, transforming once-fatal complex cardiac anomalies into manageable conditions (2, 3). As long-term survival for patients with CHD continues to rise, clinical research has increasingly shifted from prioritizing survival alone toward mitigating neurodevelopmental morbidities and optimizing long-term quality of life (2, 3).

Among the neurological complications observed in patients with CHD, clinical seizures are especially concerning given their strong association with adverse neurodevelopmental outcomes, including motor delays, language impairments, and behavioral disorders (4–6). Population-based cohort studies demonstrate that the cumulative incidence of seizures reaches approximately 5% by age 15, spanning both pre- and post-operative windows (7). Moreover, prospective evidence indicates that higher seizure incidence correlates with increased surgical complexity, prolonged hospitalizations, and the use of extracorporeal membrane oxygenation (ECMO) (8). Because continuous seizure activity induces excitotoxic neural injury and impairs early synaptic pruning, timely identification and intervention are paramount to preserving neurodevelopmental function.

Despite these recognized risks, substantial gaps remain in existing literature. First, prior epidemiological assessments have relied on bedside clinical observation or amplitude-integrated electroencephalography (aEEG), both of which have limited sensitivity in detecting subclinical events (9–11). While clinical criteria capture an initial seizure incidence of approximately 8% in hospitalized children with CHD, continuous video electroencephalographic (cEEG) monitoring reveals that the true incidence rises to 11.5%, unmasking a significant burden of subclinical, electrographic-only seizures (12). Consequently, reported seizure incidence in infants with CHD varies widely—from 5% to 30%—a discrepancy heavily confounded by differences in monitoring modalities. Second, most existing data originate from low-altitude, normoxic environments. Lanzhou, situated on the high-altitude western Loess Plateau, is characterized by lower atmospheric oxygen content and chronic hypoxic exposure. This environmental stressor contributes to a demonstrably higher incidence of CHD compared to lowland regions in China, as well as a distinct distribution of anatomical CHD subtypes. Whether these high-altitude conditions impose additive metabolic stress that exacerbates neurological vulnerability remains unclear.

To address these methodological and geographic gaps, this prospective birth cohort study was designed with three primary objectives. First, we aimed to determine the true incidence of both clinical and electrographic seizures in a high-altitude pediatric CHD cohort using comprehensive cEEG monitoring. Second, we sought to identify specific perioperative and patient-level factors associated with seizure occurrence. Finally, we evaluated the longitudinal impact of early-life seizures on multi-domain neurodevelopmental outcomes at 18 months, providing an empirical basis for targeted neuroprotective strategies in high-altitude populations.

## Methods

### Study design, participants, and procedures

This prospective birth cohort study (2018–2023) was conducted at Gansu Provincial Maternity and Child Care Hospital, enrolling pregnant women aged ≥18 years with singleton live births and intact cognitive capacity. Participants were recruited at ≥20 weeks of gestation to ensure diagnostic certainty of CHD via mid-trimester echocardiography, align with routine regional surveillance protocols, and minimize attrition bias associated with early pregnancy loss. Exclusion criteria comprised multiple gestations, stillbirths, major non-cardiac structural anomalies, severe maternal illness, or preexisting maternal or familial neurological conditions known to independently cause or trigger seizures.

**Figure 1:**
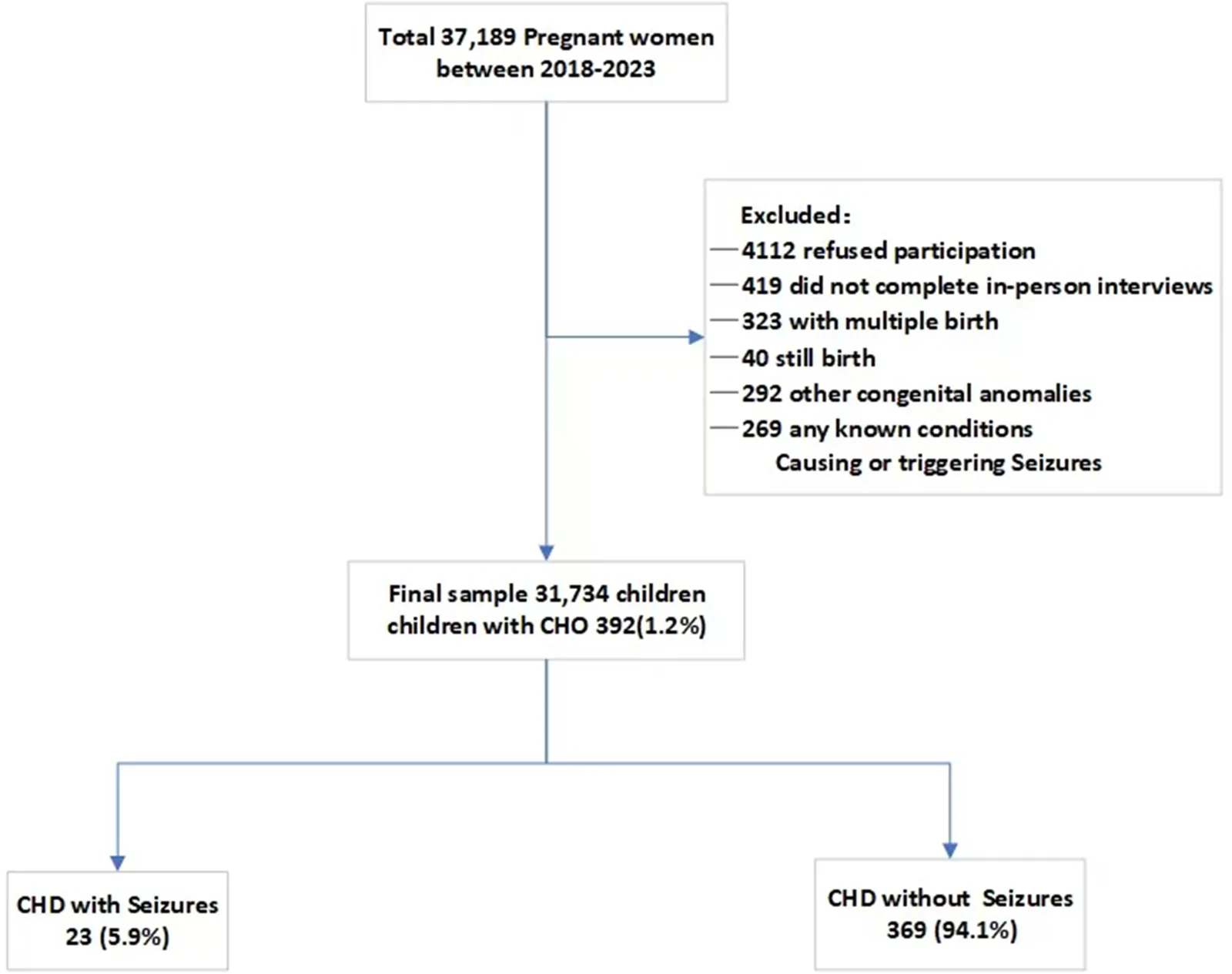
Flow Diagram.

The recruitment process is illustrated in the study flow diagram (Figure 1). Of the 37,189 women approached, 4,112 declined participation, and 419 had incomplete interviews, yielding 32,658 completed responses (87.8% response rate). Following secondary exclusions, 31,734 mother–infant dyads were included in the final cohort. Eligible women provided written informed consent after receiving a detailed study explanation. Structured face-to-face interviews were conducted 1–3 days postpartum to capture maternal and exposure variables, while perinatal outcomes were extracted and verified from electronic medical records. All 31,734 live-born infants underwent routine postnatal echocardiography screening and were followed prospectively for 18 months. The study protocol adhered to the Declaration of Helsinki and was approved by the Institutional Review Board of Gansu Provincial Maternity and Child Care Hospital (No. GSFYIRB-2018-09).

### Clinical variables

#### CHD classification

Based on postnatal echocardiography and underlying pathophysiology, infants diagnosed with CHD were stratified into four mutually exclusive categories: (1) shunt lesions with pulmonary overcirculation, (2) cyanotic lesions with persistent arterial hypoxemia, (3) left-sided lesions with impaired systemic output, and (4) lesions with single-ventricle (SV) physiology. The specific cardiac lesions encompassing each category are detailed in Supplementary Table S1. This clinical classification system was adapted from an established validation model utilized in prior large-scale cohort investigations (13).

#### Seizure and EEG definitions

Seizures occurring more than 30 days following an acute inciting event met the diagnostic criteria for epilepsy according to the International League Against Epilepsy (ILAE) guidelines (14). Specifically, epilepsy was diagnosed if a patient exhibited: (1) at least two unprovoked clinical seizures occurring > 24 h apart, (2) one unprovoked seizure with an estimated recurrence risk of at least 60% over the subsequent 10 years, or (3) a definitive electroclinical epilepsy syndrome diagnosis. Electrographic-only seizures were defined by standard neurophysiological criteria as paroxysmal EEG activity distinct from baseline background, lasting at least 10 s, and demonstrating a plausible scalp field with evolution in wave morphology, frequency, and spatial distribution (15, 16). All cEEG recordings were independently reviewed and clinically interpreted by board-certified pediatric epileptologists or clinical neurophysiologists.

#### Neurodevelopmental assessments

Neurodevelopmental outcomes were systematically evaluated at 18 months of age during structured examinations in dedicated Pediatric Neurology, Developmental Pediatrics, or Child Psychology clinics. Assessments were performed using the Bayley Scales of Infant and Toddler Development, Third Edition (Bayley-III), which provides 3 independent, standardized composite scores evaluating cognition, language, and motor skills. These composite scales are norm-referenced with a population mean of 100 and a standard deviation (SD) of 15. Consistent with diagnostic frameworks, intellectual disability (ID) was defined as concurrent impairments in cognitive functioning and adaptive behavior that originate in the early developmental period (17). Global developmental delay (GDD) was defined as significant delay across two or more independent developmental domains in children under five years of age (18).

#### Statistical analysis

All statistical analyses were performed using IBM SPSS Statistics for Macintosh (Version 25.0, Armonk, NY: IBM Corp). Before data imputation, the pattern and distribution of missing values across major clinical variables were systematically evaluated. Missingness was sparse and assumed to be missing at random (MAR), affecting <2% of data points for major baseline variables (e.g., birth weight: 1.3%; 5-minute Apgar score: 1.8%) and <5% for hospital course parameters (e.g., length of stay: 3.8%). Primary categorical predictors (such as CHD subtype, surgical history, and ECMO status) had complete data (0% missing). To preserve statistical power and sample integrity, a regression imputation approach was applied for continuous variables with missing entries. Missing values were systematically replaced with predicted values generated from linear regression equations established between the missing indicators and the observed baseline covariates.

Sample characteristics were described as means ± standard deviations (SDs) for continuous variables, and as frequencies and percentages for categorical variables. For univariable group comparisons, independent samples *t*-tests were applied to continuous variables, whereas Pearson's chi-square (*χ*2) tests were employed for categorical variables. Univariable logistic regression was used to estimate odds ratios (ORs) and 95% confidence intervals (CIs) to identify clinical factors associated with seizure occurrence in children with CHD. Due to the limited sample size of the seizure subgroup, multivariable logistic regression adjustment could not be performed. All statistical tests were two-sided, and a value of *P* < 0.05 was considered to indicate statistical significance.

## Results

### Characteristics of the cohort

A total of 37,189 pregnant women were screened, yielding a final prospective analytical cohort of 31,734 singleton live births (Table 1). Within this cohort, 392 infants (1.2%) were diagnosed with CHD. Regarding infant characteristics, slightly more than half of the infants were males (52.0%), and most were born at full term (90.4%) with normal birth weights between 2,500 and 4,000 g (88.4%). While infant sex distribution (*P* = 0.458) and prematurity rates (*P* = 0.283) did not differ significantly between groups, neonates with CHD had a significantly higher prevalence of low birth weight (< 2,500 g) than non-CHD controls (8.4% vs. 4.3%, *P* < 0.001). Maternal characteristics also differed significantly: mothers of infants with CHD were more likely to be under 25 years of age (24.2% vs. 14.3%, *P* < 0.001), have a pre-pregnancy BMI ≥ 24 kg/m^2^ (16.6% vs. 11.0%, *P* = 0.012), present with gestational diabetes (6.4% vs. 4.3%, *P* = 0.043), and lack prenatal folic acid supplementation (4.8% vs. 2.4%, *P* = 0.002).

**Table 1:** Demographic and clinical characteristics of the neonate cohort.

Characteristics in cohort	Total (*n* = 31,734)	Non-CHD (*n* = 31,342)	CHD (*n* = 392)	*P*
		*N* (%)	*N* (%)	
Infant demographic data				
Sex				
Male	16,812 (53.0)	17,081 (54.5)	221 (56.4)	0.458
Female	14,922 (47.0)	14,261 (45.5)	171 (43.6)	
Gestational age, weeks				
Preterm (<37)	3,057 (9.6)	2,789 (8.9)	41 (10.5)	0.283
Full term (≥37)	28,677 (90.4)	28,553 (91.1)	351 (89.5)	
Birth weight, g				
<2,500	2,163 (6.8)	1,347 (4.3)	33 (8.4)	**<0**.**001**
2,500–4,000	28,065 (88.4)	28,710 (91.6)	345 (88.0)	
>4,000	1,506 (4.8)	1,285 (4.1)	14 (3.6)	
Maternal & perinatal data				
Maternal age, years				
≤24	4,590 (14.5)	4,495 (14.3)	95 (24.2)	**<0**.**001**
25–29	15,547 (49.0)	15,389 (49.1)	158 (40.3)	
30–34	8,611 (27.1)	8,517 (27.2)	94 (24.0)	
≥35	2,986 (9.4)	2,941 (9.4)	45 (11.5)	
Pre-pregnancy BMI				
<18.5	6,517 (20.5)	6,542 (20.9)	75 (18.2)	**0**.**012**
18.5–23.9	21,124 (66.6)	20,668 (65.9)	256 (65.2)	
≥24	3,402 (10.7)	3,441 (11.0)	61 (16.6)	
Missing	691 (2.2)	691 (2.2)	0 (0.0)	
Multivitamin intake during pregnancy				
No	24,935 (78.6)	24,630 (78.6)	305 (77.8)	0.709
Yes	6,799 (21.4)	6,712 (21.4)	87 (22.2)	
Folic acid intake during pregnancy				
No	767 (2.4)	748 (2.4)	19 (4.8)	**0**.**002**
Yes	30,967 (97.6)	30,594 (97.6)	373 (95.2)	
Gestational diabetes				
No	30,359 (95.7)	29,992 (95.7)	367 (93.6)	**0**.**043**
Yes	1,375 (4.3)	1,350 (4.3)	25 (6.4)	
Gestational hypertension				
No	29,753 (93.8)	29,392 (93.8)	362 (92.3)	0.244
Yes	1,980(6.2)	1,950(6.2)	30 (7.7)	

Boldface *P*-values indicate statistical significance at *P* < 0.05. BMI, body mass index; CHD, congenital heart disease.

### Factors associated with seizures among patients with CHD

Among the 392 infants diagnosed with CHD, 23 developed clinical or electrographic seizures, representing an incidence rate of 5.9% within the cardiac subcohort. Table 2 summarizes the demographic, perinatal, and clinical factors associated with seizure occurrence based on univariable comparisons and unadjusted logistic regression models. All ORs reported below represent unadjusted (univariable) estimates and should not be interpreted as independent risk factors or causal effects, as multivariable adjustment was not feasible due to the limited number of seizure events.

**Table 2:** Comparison of characteristics in children with CHD presented with seizures and without seizures.

Characteristics in patients	CHD with seizures (*n* = 23)	CHD without seizures (*n* = 369)	Univariable OR (95% CI)^a^	*P*
Infant sex, female	10 (43.5)	171 (46.3)	0.879 (0.505–1.528)	0.789
Gestational age, weeks	38 ± 2.362	38.5 ± 2.118	1.561 (0.907–2.688)	0.106
Birth weight, kg	2.6 ± 1.076	3.2 ± 1.175	1.024 (1.001–1.058)	0.037
APGAR score at 5 min	9 (5–9)	9 (6–9)	0.898 (0.688–1.207)	0.579
CHD types				
Shunt CHD	3 (13.0)	273 (74.0)	–	**<0**.**001**^b^
Cyanotic CHD	6 (26.1)	42 (11.4)		
Left side CHD	6 (26.1)	35 (9.5)		
Single ventricle physiology	8 (34.7)	20 (5.4)		
Surgery				
No	4 (17.4)	270 (73.2)	–	**<0**.**001**^b^
Yes	19 (82.6)	99 (26.8)		
Need for ECMO (pre/op/post)				
No	19 (82.6)	364 (98.6)	–	**<0**.**001**^b^
Yes	4 (17.4)	5 (1.4)		
Hospital length of stay, days	43 ± 13.752	21 ± 11.329	1.484 (1.167–1.881)	**<0**.**001**

Boldface *P*-values indicate statistical significance at *P* < 0.05. APGAR, appearance, pulse, grimace, activity, and respiration; CHD, congenital heart disease; CI, confidence interval; ECMO, extracorporeal membrane oxygenation; OR, odds ratio; pre/op/post, preoperative, perioperative, or postoperative; SD, standard deviation.

a Unadjusted odds ratios from univariable analysis. Multivariable adjustment was not performed due to small sample size; estimates reflect bivariate associations rather than independent risk predictors.

b Pearson's Chi-square test.

While demographic variables—including sex, gestational age, and 5-minute Apgar scores—were comparable between groups, lower birth weight was significantly associated with increased seizure risk (OR = 1.02, 95% CI: 1.00–1.05). Seizure occurrence was strongly linked to cardiac lesion anatomy and perioperative complexity; infants with complex structural defects—specifically SV physiology (34.7% vs. 5.4%), cyanotic lesions (26.1% vs. 11.4%), and left-sided obstructive defects (26.1% vs. 9.5%)—were disproportionately affected compared to those with simple left-to-right shunts (*P* < 0.001). Furthermore, seizures were highly correlated with intensive clinical interventions, including surgical intervention (*P* < 0.001) and ECMO support (*P* < 0.001). Seizure incidence was significantly higher among infants undergoing surgical repair compared to non-surgical cases [16.1% [19/118] vs. 1.5% [4/274], *P* < 0.001], with surgical patients accounting for 82.6% (19/23) of all seizure cases. Consequently, infants experiencing seizures had significantly prolonged hospital stays compared to seizure-free counterparts (OR = 1.48; 95% CI 1.17–1.89).

### Seizure semiology and EEG characteristics in patients with CHD

The clinical semiology and EEG phenotypes of the 23 infants with CHD who developed seizures are detailed by underlying cardiac physiology in Table 3. Regarding seizure semiology, focal seizures represented the predominant semiology across the subcohort, accounting for 56.5% (*n* = 13/23) of all events. This predominance was particularly pronounced among patients with SV physiology, of whom 75.0% (*n* = 6/8) exhibited focal semiology. Generalized tonic-clonic (GTC) seizures were the second most frequent phenotype, occurring in 26.1% (*n* = 6/23) of cases with a relatively uniform distribution across shunt, cyanotic, and left-sided obstructive lesions. Myoclonic seizures (*n* = 2, 8.7%) and subclinical, electrographic-only seizures (*n* = 2, 8.7%) occurred less frequently, with electrographic-only events isolated exclusively to cyanotic and left-sided CHD subtypes.

**Table 3:** Seizure semiology and electroencephalographic characteristics across CHD pathophysiological subtypes (*n* = 23).

Characteristics of seizure	Total	Shunt CHD	Cyanotic CHD	Left-sided CHD	Single ventricle physiology
Number of patients	23	3	6	6	8
Seizure semiology					
GTC	6	1	2	2	1
Myoclonia	2	0	0	1	1
Focal	13	2	3	2	6
Electrographic	2	0	1	1	0
EEG					
Continuous, slow, generalized	4	0	3	1	0
Generalized/multifocal spikes, sharps, or spike wave complexes	8	3	1	2	2
Focal discharges	7	0	1	1	5
Background suppression	2	0	0	1	1

Data are presented as n. CHD, congenital heart disease; EEG, electroencephalograph; GTC, generalized tonic-clonic.

Neurophysiological classification of EEG tracings revealed distinct background and epileptiform patterns that aligned with anatomical subgroups. Generalized or multifocal paroxysms (including spikes, sharp waves, or spike-wave complexes) were identified in 34.8% (*n* = 8/23) of seizure cases, including all patients within the shunt CHD category (100.0%). Conversely, isolated focal discharges were observed in 30.4% (*n* = 7/23) of cases and were heavily concentrated among infants with SV physiology (*n* = 5/7). Severe background abnormalities were also observed, characterized by continuous generalized slowing in 17.4% (*n* = 4/23) of cases—predominating in cyanotic CHD (50.0%)—and background suppression in 8.7% (*n* = 2/23), restricted to infants with left-sided lesions and SV anatomy.

### Neurodevelopmental outcomes at 18 months

Of the original 392 infants with CHD, 358 completed formal neurodevelopmental assessments at 18 months of age (91.3% retention rate), comprising 19 children in the early seizure group and 339 in the seizure-free control group. Loss to follow-up was primarily attributed to parental refusal, commonly cited as the perception of normal neurodevelopment. Comparative analyses of multi-domain functional performance and clinical diagnoses are summarized in Table 4.

**Table 4:** Neurodevelopmental outcomes at 18 months between CHD patients with and without seizures (*n* = 358).

Outcome parameter	CHD with seizures (*n* = 19)	CHD without seizures (*n* = 339)	t/*χ*2	*P*
Bayley-III Scales, Mean ± SD				
Cognitive scale	91 ± 20.176	95 ± 19.324	1.043	0.254
Language scale	78 ± 27.329	89 ± 21.762	4.233	0.062
Motor scale	82 ± 19.654	100 ± 32.719	3.923	**0**.**038**
Clinical diagnoses, *n* (%)				
ID or GDD	14 (73.7)	97 (28.6)	24.564	**<0**.**001**
Subsequent epilepsy	3 (15.8)	0 (0.0)	–	–

Boldface *P*-values indicate statistical significance at *P* < 0.05. Bayley-III, Bayley Scales of Infant Development, Third Edition; CHD, congenital heart disease; GDD, global developmental delay; ID, intellectual disability; SD, standard deviation.

Standardized testing using the Bayley-III framework revealed that infants with a history of early-life seizures scored significantly lower on the Motor scale at 18 months than seizure-free controls (82.1 ± 19.7 vs. 100.0 ± 32.7, *P* = 0.038). A downward trend was also observed on the Language scale (78.0 ± 27.3 vs. 89.0 ± 21.8, *P* = 0.062), whereas cognitive composite scores did not differ significantly between groups (*P* = 0.254). Regarding categorical clinical diagnoses, early-life seizures were strongly associated with adverse developmental milestones; the combined prevalence of ID or GDD was more than twice as high in the seizure group as in controls (73.7% vs. 28.6%; *P* < 0.001). Furthermore, chronic epilepsy was isolated exclusively to the early seizure cohort, with 15.8% (*n* = 3/19) progressing to post-neonatal epilepsy by 18 months, compared to 0% in the control group.

## Discussion

### Incidence and factors associated with CHD at high altitude

This prospective cohort study established an infant CHD incidence of 1.2% within the western Loess Plateau region of Lanzhou, China. To contextualize our findings, we compared our CHD incidence with data reported from other high-altitude populations. He et al. (19) reported a CHD prevalence of approximately 1.4% among school children in Qinghai Province (average altitude >3,000 m), which is comparable to our Lanzhou cohort (altitude ∼1,500–2,000 m). Studies from Tibet (average altitude > 3,500 m) have reported CHD prevalence of 1.8%–2.4%, substantially exceeding our rate (20, 21). Data from other international high-altitude populations, including the Andes (Peru, Bolivia) and the Ethiopian highlands, also demonstrate elevated CHD prevalence at higher altitudes, though direct comparison is limited by differences in screening protocols and case definitions (22). Our Lanzhou cohort is at an intermediate altitude, which may explain why our incidence (1.2%) is higher than lowland estimates (∼0.6%–0.8%) (23) but lower than extreme-altitude estimates from Tibet and Qinghai (19–21). This gradient supports the hypothesis that chronic hypoxia is a dose-dependent factor associated with CHD development.

Previous studies have established altitude as an independent risk factor for CHD, highlighting profound geographical variation in congenital cardiac susceptibility (19, 24). The atmospheric physics of high-altitude environments offers a clear physiological mechanism for these variations: decreased barometric pressure reduces ambient oxygen availability, leading to systemic maternal and fetal hypoxemia (25, 26). This reduced oxygen tension directly impairs the physiological mechanisms governing spontaneous ductus arteriosus closure post-birth. Exacerbated by hypoxemia-induced hyperhemoglobinemia and elevated pulmonary arterial pressure, persistent hypoxia can delay or prevent defect closure, thereby serving as a key driver of postnatal structural CHD. Additionally, our study identified maternal age under 25 years, elevated pre-pregnancy BMI (≥ 24 kg/m^2^), gestational diabetes, and low birth weight as factors associated with higher CHD prevalence, whereas maternal folic acid supplementation was associated with lower prevalence—findings consistent with previous literature (27, 28).

### Mechanisms of seizure susceptibility

Among infants diagnosed with CHD, the incidence of early-life clinical or electrographic seizures was 5.9%, a rate highly consistent with current international literature (29–31). This figure aligns with reported rates from major sea-level pediatric cardiac centers (4%–10%) (13, 32, 33). While dedicated seizure data in high-altitude CHD cohorts remain sparse, chronic baseline hypoxemia at moderate-to-high altitudes may exacerbate perioperative cerebral vulnerability, underscoring the critical need for continuous neuromonitoring in these populations. However, direct comparisons with previous large multicenter studies require careful consideration of methodological differences. Multicenter registries employing universal, long-term continuous video-EEG monitoring routinely report higher seizure detection rates—particularly of subclinical electrographic seizures—than centers that rely primarily on clinical detection or selective EEG screening. Furthermore, variations in surgical management (e.g., deep hypothermic circulatory arrest vs. low-flow cardiopulmonary bypass) across international centers significantly influence the frequency of acute post-surgical seizures.

Intriguingly, infants presenting with cyanotic CHD exhibited a lower overall incidence of acute seizures compared to other CHD categories, consistent with prior studies (34). This clinical phenomenon is supported by foundational animal models demonstrating that acute systemic hypotension and subsequent ischemic-reperfusion injury exert a far more disruptive impact on neonatal electrocortical background activity than isolated hypoxemia (35). Physiologically, this observation aligns with the concept of chronic hypoxia-induced preconditioning. As demonstrated in young rodent seizure models by Zhen et al. (36) and Xie et al. (37), sustained exposure to high-altitude hypoxia elevates the baseline seizure threshold and attenuates hippocampal neuronal apoptosis following secondary acute insults. This adaptive metabolic preconditioning likely shields cyanotic infants from severe acute electrographic paroxysms despite persistent systemic arterial desaturation.

Conversely, acute seizure presentation was associated with the cumulative stresses of surgical complexity and mechanical circulatory support. Infants requiring cardiopulmonary bypass surgeries or perioperative ECMO support demonstrated exceptionally high seizure rates. Infants undergoing cardiac surgery exhibited a markedly elevated risk of clinical seizures compared to those managed non-surgically. Cardiac surgical procedures in neonates and infants often involve exposure to cardiopulmonary bypass, hypothermic arrest, microembolization, and systemic inflammatory responses, which can induce focal cerebral ischemia or acute hypoxic-ischemic injury (32, 38). Combined with baseline cerebral dysmaturation and chronic preoperative hypoxia common in complex CHD, these intraoperative and postoperative stressors significantly lower the seizure threshold (32, 38). However, the precise timing of seizure onset relative to surgery (preoperative vs. postoperative) was not systematically recorded in our registry, preventing a direct comparison of pre- and post-surgical incidence rates.

The finding that receiving ECMO support was associated with higher odds of seizure is consistent with other studies (39, 40). This heightened vulnerability reflects a multi-hit mechanism of neurologic injury wherein cannula-mediated thromboembolism, altered pulsatile cerebral perfusion, and systemic inflammatory cascades synergistically disrupt the fragile neonatal blood-brain barrier. Consequently, the presence of seizure activity served as a marker for heightened clinical complexity, showing a robust association with a doubling of institutional lengths of stay. It is critical to interpret the reported odds ratios with caution. Because the small number of seizure events precluded multivariable regression analysis, these estimates represent unadjusted bivariate associations rather than independent risk factors. Consequently, these findings demonstrate statistical correlation rather than direct causality, as unmeasured perioperative and physiological confounders may account for the observed relationships.

### Subtype-specific mechanisms of neurological injury and seizure phenotypes

An important observation in our study was the distinct pattern of seizure semiology and EEG features across CHD pathophysiological subtypes (Table 3). Infants with cyanotic CHD presented predominantly with generalized background slowing, reflecting diffuse, symmetric cerebral hypoxemia and altered global cerebral energy metabolism (41). Beyond general focal activity, a unique cluster of focal autonomic impaired awareness seizures —characterized by prominent ictus emesis (vomiting) alongside consciousness impairment—manifested in 46% of the SV subgroup, particularly among those with hypoplastic left heart syndrome (HLHS). Furthermore, EEG tracings in these infants uniquely revealed sleep-activated epileptic discharges localized predominantly to the occipital channels.

We hypothesize that these subtype-specific profiles reflect discrete underlying mechanisms of brain injury—such as global hypoxic-ischemic encephalopathy in cyanotic lesions versus localized structural insults in SV physiology (42). Severe left-sided obstructive lesions like HLHS alter retrograde cerebral blood flow dynamics during critical windows of third-trimester neurogenesis, causing subtle microstructural cortical insults within posterior watershed territories (7). Following birth, the acute metabolic stress of stage-one palliative surgeries—which involve prolonged cardiopulmonary bypass, deep hypothermic arrest, and selective regional perfusion—unmasks this localized hyperexcitability and heightens the risk of focal cerebral microembolism (32). This combination ultimately generates an electroclinical phenotype that targets subcortical emesis networks, mimicking self-limited autonomic epilepsy.

### Long-term neurodevelopmental trajectories

Regarding neurodevelopmental trajectories, our finding that 30.1% of infants with CHD presented with ID or GDD aligns closely with long-term multicenter benchmarks, such as the Boston Circulatory Arrest Study and related registry data (31, 43). While multicenter cohorts similarly demonstrate that acute seizures and stroke independently predict adverse neurodevelopmental scores, single-center cohorts may display variability in functional outcomes driven by local referral patterns, differences in postoperative intensive care protocols, and varying access to early intervention programs. Crucially, the co-occurrence of early-life seizures profoundly compounded these adverse neurodevelopmental milestones, significantly increasing the incidence of ID or GDD compared to seizure-free counterparts (*P* < 0.001). Standardized multi-domain neurodevelopmental assessment using the Bayley Scales substantiated these functional deficits, revealing significantly lower motor scores in the seizure subgroup compared with those without seizures (*P* = 0.038). This tight clinical clustering of chronic epilepsy with pervasive cognitive and motor delays strongly suggests that the development of epilepsy in children with CHD is multifactorial. In a subset of patients, these neurological phenotypes may be influenced more by co-occurring conditions rather than by the presence of CHD itself.

### Strengths and limitations

The main strength of this study lies in its prospective evaluation of an exceptionally large birth cohort within the unique, high-altitude environment of the western Loess Plateau, offering an unbiased assessment of an underrepresented pediatric population. Our study minimizes selection bias by encompassing the complete spectrum of CHD phenotypes associated with seizures while rigorously excluding non-cardiac genetic and syndromic etiologies of neonatal epilepsy. Furthermore, the systematic integration of continuous video-EEG monitoring enabled the precise capture of subclinical, electrographic-only seizures that would otherwise remain undetected by bedside clinical observation alone.

Our study has several limitations that must be acknowledged. First, the single-center study design and regional recruitment may limit generalizability to broader populations, highlighting the need for future investigations that utilize large, multicenter international registries. Second, the small sample size of seizures (*n* = 23) precluded the use of multivariate regression modeling. As a result, reported odds ratios reflect unadjusted associations rather than independent risk predictors, and causal inferences cannot be drawn. Prospective multicenter studies with larger cohorts are needed to perform multivariable risk modeling. Third, our observation of a candidate electroclinical cluster—focal autonomic impaired-awareness seizures with occipital EEG discharges in SV and HLHS infants—remains preliminary and hypothesis-generating due to small subgroup numbers, requiring validation in future large-scale cohorts employing long-term continuous video-EEG monitoring. Fourth, brain magnetic resonance imaging was not performed in all participants, which may have led to an underestimation of subtle, subclinical structural brain injuries; future study protocols should systematically integrate standardized perioperative brain MRI alongside continuous EEG. Finally, the absence of routine genetic screening and next-generation sequencing left underlying pathogenic copy-number variations unmeasured, underscoring the need for future research to incorporate systematic genetic profiling to better delineate the complex interplay among genetic etiologies, cardiac defects, and post-surgical seizure vulnerability.

## Conclusions

This prospective cohort study in a high-altitude population found a 1.2% infant CHD incidence, with 5.87% of affected infants experiencing early-life seizures. Factors associated with seizure occurrence included lower birth weight, SV physiology, surgery, and ECMO. Notably, we observed a potential clinical cluster of focal autonomic impaired awareness seizures with sleep-activated occipital EEG discharges, particularly among infants with SV physiology and HLHS. While these preliminary findings based on an observational study offer a potential framework for risk stratification and neuroprotective planning, larger multicenter studies are needed to validate this candidate electroclinical phenotype.

## Data Availability

The original contributions presented in the study are included in the article/Supplementary Material, further inquiries can be directed to the corresponding author.
