# Supplementary material for: Seizures in congenital heart disease: risks, outcomes, and birth cohort data from China
Source: Front Pediatr. 2026 Aug 25;14:1936201. doi: 10.3389/fped.2026.1936201 (PMC13550128; doi:10.3389/fped.2026.1936201)
Supplement: Supplementary file 1 [file Table1.docx]

**Supplementary Table 1. ICD 10 Used to Identify Congenital Heart Disease Categorized by Nature of Clinical Presentation**

| **Congenital Heart Disease** | ***ICD-10* codes** |
| --- | --- |
| Category A: Shunt lesions: pulmonary overcirculation | |
| Double outlet right ventricles | Q20.1 |
| Ventricular septal defect (Isolated) | Q21.0 |
| Atrial septal defect (Isolated) | Q21.1 |
| Atrio-ventricular septal defect | Q21.2 |
| Partial/complete atrio-ventricular defect | Q21.20 |
| Partial atrio-ventricular septal defect | Q21.21 |
| Transitional atrio-ventricular septal defect | Q21.22 |
| Complete atrio-ventricular septal defect | Q21.23 |
| Aortopulmonary septal defect | Q21.4 |
| Category B: Cyanotic lesions: persistent arterial hypoxemia | |
| Common arterial trunk | Q20.0 |
| Transposition of the great arteries | Q20.3 |
| Tetralogy of Fallot | Q21.3 |
| Pulmonary valve atresia | Q22.0 |
| Pulmonary valve stenosis | Q22.1 |
| Ebstein anomaly | Q22.5 |
| Total anomalous pulmonary venous return | Q26.2 |
| Category C: Left-sided lesions: impaired systemic output | |
| Aortic valve stenosis | Q23.0 |
| Aortic valve insufficiency | Q23.1 |
| Mitral valve stenosis | Q23.2 |
| Mitral valve insufficiency | Q23.3 |
| Cor Triatriatum | Q24.2 |
| Congenital subaortic stenosis | Q24.4 |
| Coarctation of the aorta | Q25.1 |
| Interrupted aortic arch | Q25.21 |
| Supravalvar aortic stenosis | Q25.3 |
| Aortic hypoplasia | Q25.42 |
| Category D: Single ventricle physiology | |
| Double inlet ventricle | Q20.4 |
| Tricuspid atresia | Q22.4 |
| Pulmonary artery atresia | Q25.5 |
| Hypoplastic right heart syndrome | Q22.6 |
| Hypoplastic left heart syndrome | Q23.4 |
| Atresia of the aorta | Q25.2 |

**ICD-10 indicates International Classification of Diseases, Tenth Revision[13].*
